# Genetic and phenotypic analysis of the pathogenic potential of two novel *Chlamydia gallinacea* strains compared to *Chlamydia psittaci*

**DOI:** 10.1038/s41598-021-95966-9

**Published:** 2021-08-13

**Authors:** Marloes Heijne, Martina Jelocnik, Alexander Umanets, Michael S. M. Brouwer, Annemieke Dinkla, Frank Harders, Lucien J. M. van Keulen, Hendrik Jan Roest, Famke Schaafsma, Francisca C. Velkers, Jeanet A. van der Goot, Yvonne Pannekoek, Ad P. Koets

**Affiliations:** 1 Department of Bacteriology and Epidemiology, Wageningen Bioveterinary Research, Lelystad, The Netherlands; 2 Genecology Research Centre, University of the Sunshine Coast, Sippy Downs, Australia; 3 Department of Infection Biology, Wageningen Bioveterinary Research, Lelystad, The Netherlands; 4 Department of Population Health Sciences, Faculty of Veterinary Medicine, Utrecht University, Utrecht, The Netherlands; 5 Department of Diagnostics and Crisis Organisation, Wageningen Bioveterinary Research, Lelystad, The Netherlands; 6 Department of Medical Microbiology, Amsterdam UMC, University of Amsterdam, Amsterdam, The Netherlands; 7 Present Address: Directorate Animal Supply Chain and Animal Welfare, Ministry of Agriculture, Nature and Food Quality, The Hague, The Netherlands; 8 Present Address: Department of Healthy Living, TNO, Zeist, The Netherlands

**Keywords:** Biological techniques, Genetics, Microbiology, Diseases

## Abstract

*Chlamydia gallinacea* is an obligate intracellular bacterium that has recently been added to the family of *Chlamydiaceae*. *C. gallinacea* is genetically diverse, widespread in poultry and a suspected cause of pneumonia in slaughterhouse workers. In poultry, *C. gallinacea* infections appear asymptomatic, but studies about the pathogenic potential are limited. In this study two novel sequence types of *C. gallinacea* were isolated from apparently healthy chickens. Both isolates (NL_G47 and NL_F725) were closely related to each other and have at least 99.5% DNA sequence identity to *C. gallinacea* Type strain 08-1274/3. To gain further insight into the pathogenic potential, infection experiments in embryonated chicken eggs and comparative genomics with *Chlamydia psittaci* were performed. *C. psittaci* is a ubiquitous zoonotic pathogen of birds and mammals, and infection in poultry can result in severe systemic illness. In experiments with embryonated chicken eggs, *C. gallinacea* induced mortality was observed, potentially strain dependent, but lower compared to *C. psittaci* induced mortality. Comparative analyses confirmed all currently available *C. gallinacea* genomes possess the hallmark genes coding for known and potential virulence factors as found in *C. psittaci* albeit to a reduced number of orthologues or paralogs. The presence of potential virulence factors and the observed mortality in embryonated eggs indicates *C. gallinacea* should rather be considered as an opportunistic pathogen than an innocuous commensal.

## Introduction

*Chlamydiaceae* are a family of obligate intracellular bacteria containing one genus and 14 species, and comprising human and animal pathogens. In birds, infections are caused by *Chlamydia psittaci* or more recently recognized species such as *C. gallinacea*^1^. *C. psittaci* is zoonotic and has been reported worldwide in more than 465 bird species belonging to at least 30 orders^2^. Most human infections have been linked to contact with birds or their environments^3^. *C. gallinacea* is mainly detected in poultry with reports from almost all continents^4–6^. *C. gallinacea* has incidentally been found in wild birds and cattle as a possible result of infection spill-over^7,8^. Possible zoonotic transmission of *C. gallinacea* has been considered but could neither be confirmed nor ruled out in slaughterhouse workers that developed pneumonia after they were exposed to *C. gallinacea* infected poultry^9^.

Infections with *C. psittaci* in birds are often asymptomatic, but can result in localized syndromes (e.g., conjunctivitis) or severe systemic illness. Chlamydial strain, avian host, host age and (environmental) stressors are important factors in the occurrence and severity of clinical signs^3^. Studies investigating the pathogenesis of *C. gallinacea* in birds are currently limited. As yet, clinical signs of disease in *C. gallinacea* infections have not been reported in observational field studies^4,9,10^. Under experimental conditions it has been demonstrated that infection in broilers results in reduced weight gain^4^. In a transmission study, *C. gallinacea* was mainly present in rectal and cloacal samples without clinical signs of disease and transmission occurred via the faecal-oral route^11^. Thereby, at present *C. gallinacea* is considered a rather non-pathogenic species.

Molecular studies using outer membrane protein A (*ompA*) genotyping or Multi Locus Sequence Typing (MLST) showed *C. gallinacea* is diverse, with at least 13 different *ompA* types and 15 different sequence types (ST) in 25 strains^4,12^. Fine detail comparative genomics revealed that the *C. gallinacea* genome is conserved, syntenic and compact, but possesses the hallmark of chlamydial specific virulence factors: inclusion membrane (Inc) proteins, polymorphic membrane proteins (Pmps), a Type III Secretion System (T3SS), a plasticity zone with a cytotoxin (*tox*) gene, and the chlamydial virulence plasmid^12,13^. Whether this genetic diversity and the presence of chlamydial virulence genes contributes to the pathogenicity of *C. gallinacea* remains a question, as clinical disease in infected chickens has not been reported in the limited number of field and experimental studies.

The aim of this study was to investigate the pathogenicity of two novel *C. gallinacea* strains by comparing them to a virulent *C. psittaci* strain using an in vivo infection model in embryonated chicken eggs and performing comparative genomics with inter- and intra-species genomes. In the eggs, *C. gallinacea* induced mortality was observed, but to a lower extent than *C. psittaci* induced mortality. Comparative genomics showed that both novel *C. gallinacea* isolates possess the hallmark genes coding for known and potential virulence factors as found in *C. psittaci*, albeit to a reduced number of orthologs or alleles. The current results indicate *C. gallinacea* should be considered as an opportunistic pathogen rather than an innocuous commensal.

## Results

### Isolation and pathology of *C. gallinacea* NL_G47 and NL_F725 in embryonated chicken eggs

Layer flocks at the Faculty of Veterinary Medicine in Utrecht, the Netherlands were monitored for the presence of *C. gallinacea* to isolate Dutch field strains. In these flocks, *C. gallinacea* strain NL_G47 could be isolated from a caecal scraping sample collected in January 2018 from a 40-week old clinically healthy layer hen. *C. gallinacea* strain NL_F725 could be isolated from a caecal suspension sample collected in August 2017 from a 34-week old layer hen. Both hens originated from different flocks, but were housed at the same location at different time points. About one month before the *C. gallinacea* positive caecal samples were collected, both flocks tested PCR positive for *C. gallinacea* in environmental boot sock samples as shown in the timeline of Supplementary Fig. S1. *C. gallinacea* positivity in the flock from strain NL_F725 preceded a coinciding Infectious Laryngotracheitis (ILT) infection. To prevent further spread of ILT the flock had to be culled. Background data of the flocks are added to Supplementary Data S1.

*C. gallinacea* NL_G47 and NL_F725 were isolated in the yolk sac of embryonated specific-pathogen-free (SPF) chicken eggs and replication was confirmed with positive immunofluorescence of the yolk sac membrane (see Supplementary Fig. S2) and a positive *Chlamydiaceae* PCR targeting the 23S rRNA gene. With the isolation of NL_G47 in the yolk sac of embryonated eggs, mortality was observed at day 10 after inoculation (incubation day 16) and at day 6 (incubation day 12) in the second passage. At primary isolation of NL_F725 no mortality of the embryos was observed, but eggs were harvested before day 10 after inoculation (day 8 after inoculation, incubation day 14) for logistical reasons. With the second passage of NL_F725, mortality of the embryos was observed at day 6 or day 7 after inoculation (incubation day 12 or 13). Based on egg candling, congestion of the blood vessels was observed prior to mortality of the embryos. At harvest the embryos were deep red (rubor), showed cyanotic toes and haemorrhaging of the skin (Supplementary Fig. S2).

**Figure 1:**
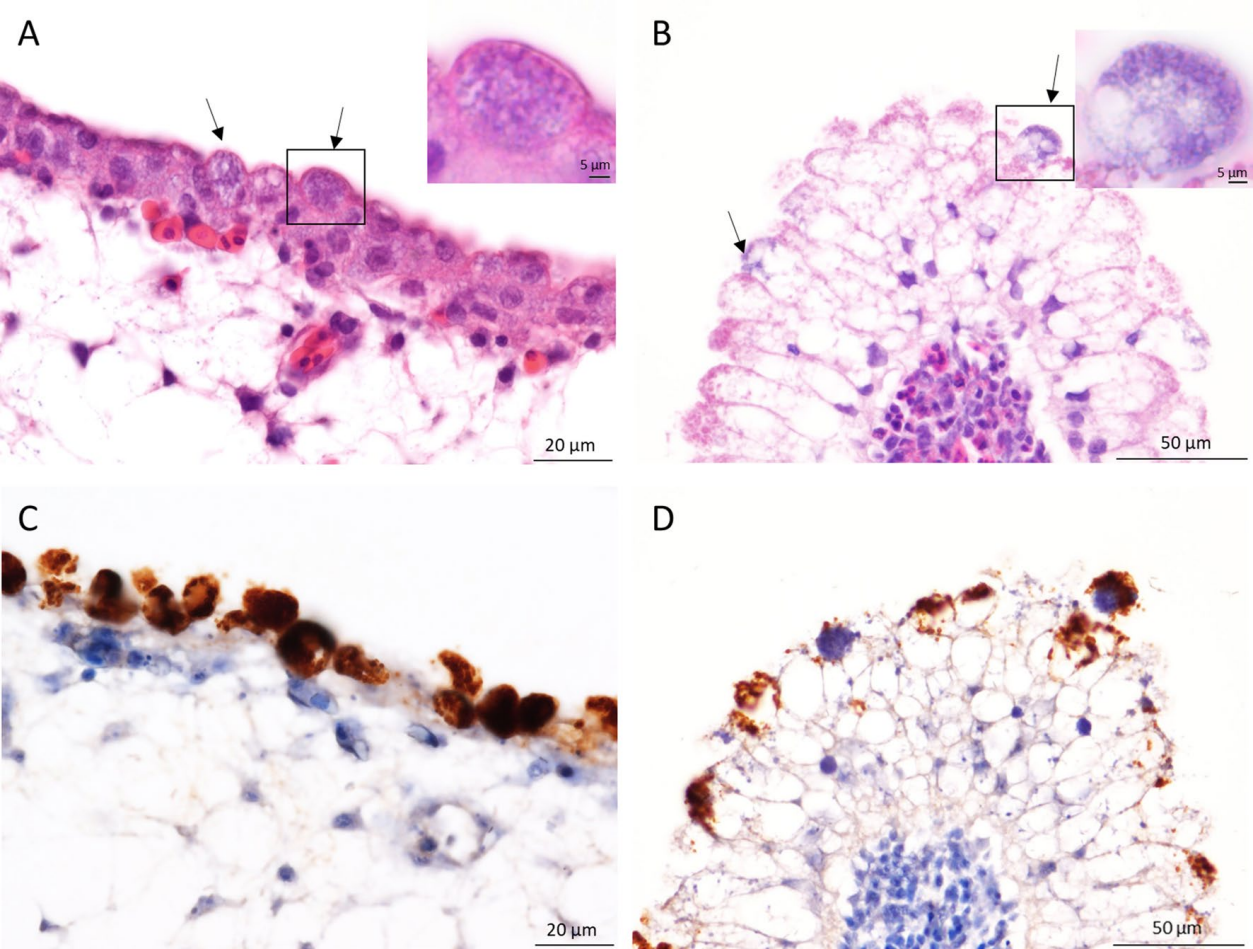
Chorioallantoic membrane and yolk sac membrane of 10 days embryonated eggs infected with NL_G47. Intracellular inclusions (arrows) in the epithelial cells of the chorioallantoic membrane (**A**) and yolk sac membrane (**B**). Inset: higher magnification showing the granular basophilic inclusions in the HE staining. Positive immunolabelling of the intracellular inclusions for chlamydial antigen in the chorioallantoic membrane (**C**) and yolk sac membrane (**D**). Sections were photographed with an Olympus BX51 microscope equipped with a high-resolution digital camera and using Olympus’ cellSens software.

To investigate any histological lesions NL_G47 infected eggs were harvested at day 10 of incubation when anomalies of the vessels were observed with candling. Granular basophilic intracellular inclusions were seen in the epithelial cells of both the chorioallantoic membrane and the yolk sac membrane (Fig. 1A,C). These intracellular inclusions were strongly positive for chlamydial antigen labelling (Fig. 1B,D).

Primary isolation and propagation of *C. gallinacea* NL_G47 and NL_F725 in Buffalo Green Monkey (BGM) cells initially failed, but after three passages in eggs the strains could be propagated in BGM cells.

### Assessment of virulence of *C. gallinacea* in embryonated eggs

**Figure 2:**
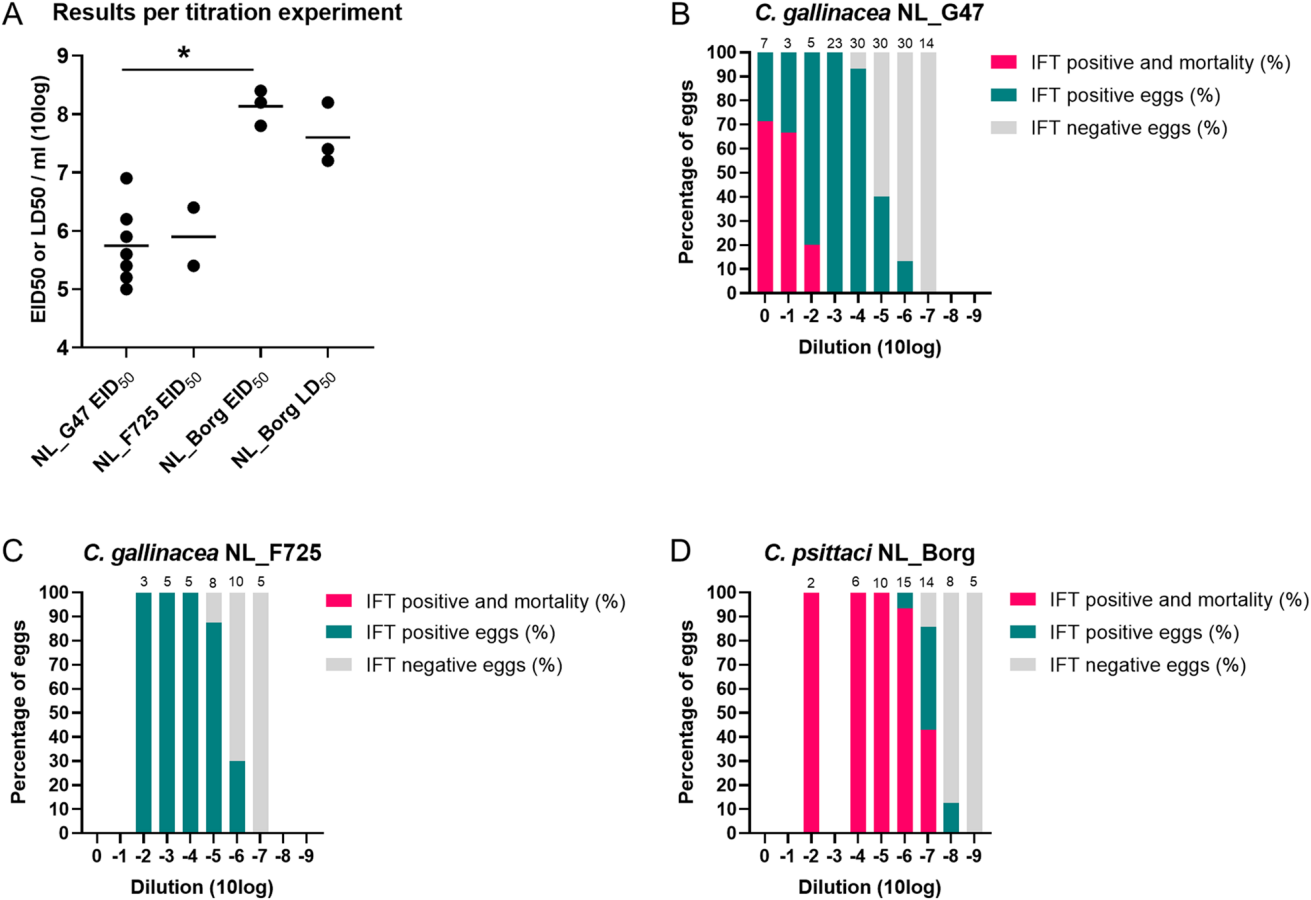
Assessment of virulence of *C. gallinacea* in embryonated eggs. (**A**) The 50% egg infective dose 50 (EID_50_) of *C. gallinacea* NL_G47, NL_F725 and *C. psittaci* NL_Borg based on IFT of the yolk sac. The difference between EID_50_ of NL_G47 and NL_Borg was significantly different (*, *P* < 0.05, Wilcoxon–Mann–Whitney test). For *C. psittaci* NL_Borg the 50% lethal dose (LD_50_) was also calculated. The median EID_50_ or LD_50_ of the experiments is indicated with a bar. (**B**–**D**) Depict the cumulative results of the separate titration experiments per *Chlamydia* strain. Per dilution, the percentage of eggs that was positive for *Chlamydia* in the immunofluorescence test (IFT) with mortality, IFT positive without mortality and IFT negative are shown. The total number of eggs per dilution are presented at the top of every bar. These data are also included in Supplementary Table S1. The figure was created in GraphPad Prism 9.0.0.

Titration experiments in embryonated chicken eggs were performed to quantify the infectious dose and gain further insight into the pathogenic potential of the novel isolates compared to *C. psittaci*. Ten-fold serial dilutions of third passage yolk sac cultures of *C. gallinacea* NL_G47 and NL_F725, and *C. psittaci* NL_Borg, were used to calculate the 50% egg infective dose (EID_50_) based on positivity in the immunofluorescence test (IFT) of the yolk sac membrane (with or without mortality of the eggs). The experiments were repeated seven times for NL_G47 with a median EID_50_ of 10^5.6^, two times for NL_F725 with a median EID_50_ of 10^5.9^ and three times for NL_Borg with a median EID_50_ 10^8.2^. All negative control eggs that were inoculated with Dulbecco’s Phosphate Buffered Saline (DPBS), remained viable until harvesting and tested *Chlamydia* negative by the IFT, except in one experiment with NL_G47 where aspecific mortality was observed in two of four eggs within three days after inoculation. As shown in Fig. 2A, the EID_50_ of *C. psittaci* strain NL_Borg was significantly higher than the EID_50_ (*P* < 0.05, Wilcoxon–Mann–Whitney test) of *C. gallinacea* NL_G47. The EID_50_ of NL_F725 was in the same range as the EID_50_ of NL_G47, but could not be statistically assessed due the low number of observations.

For *C. psittaci* NL_Borg the 50% lethal dose (LD_50_) could also be calculated from the experiments with a median LD_50_ of 10^7.4^. The LD_50_ of the experiments with *C. psittaci* NL-Borg showed overlap with the calculated EID_50_ (Fig. 2A). The LD_50_ from the experiments with *C. gallinacea* NL_G47 and NL_F725 could not be calculated, because the number of eggs in the dilutions with observed mortality was too low to calculate the LD_50_. To get further insight into differences in mortality and infectivity between *C. gallinacea* and *C. psittaci*, the data from all separate experiments were merged into one dataset (see Supplementary Table S1).

The percentage of eggs that was IFT positive with mortality, IFT positive without mortality and IFT negative is shown per dilution and per *Chlamydia* strain (Fig. 2B–D). For *C. gallinacea* strain NL_G47, mortality was observed until the 10^−2^ dilution and IFT positivity until the 10^−6^ dilution (Fig. 2B). For *C. gallinacea* strain NL_F725 no mortality was observed in the dilutions that were tested (from 10^−2^ until 10^−7^), but IFT positivity was seen until the 10^−6^ dilution similar to *C. gallinacea* NL_G47 (Fig. 2C). For *C. psittaci* strain NL_Borg, mortality was observed until dilution 10^−7^ and IFT positivity until 10^−8^ (Fig. 2D). These results indicate mortality in the *C. psittaci* infected eggs was relatively higher than in the *C. gallinacea* infected eggs and there might be a difference in mortality between *C. gallinacea* strains, although the number of observations was low.

### General characteristics of the genome sequences of Dutch *C. gallinacea* isolates

**Figure 3:**
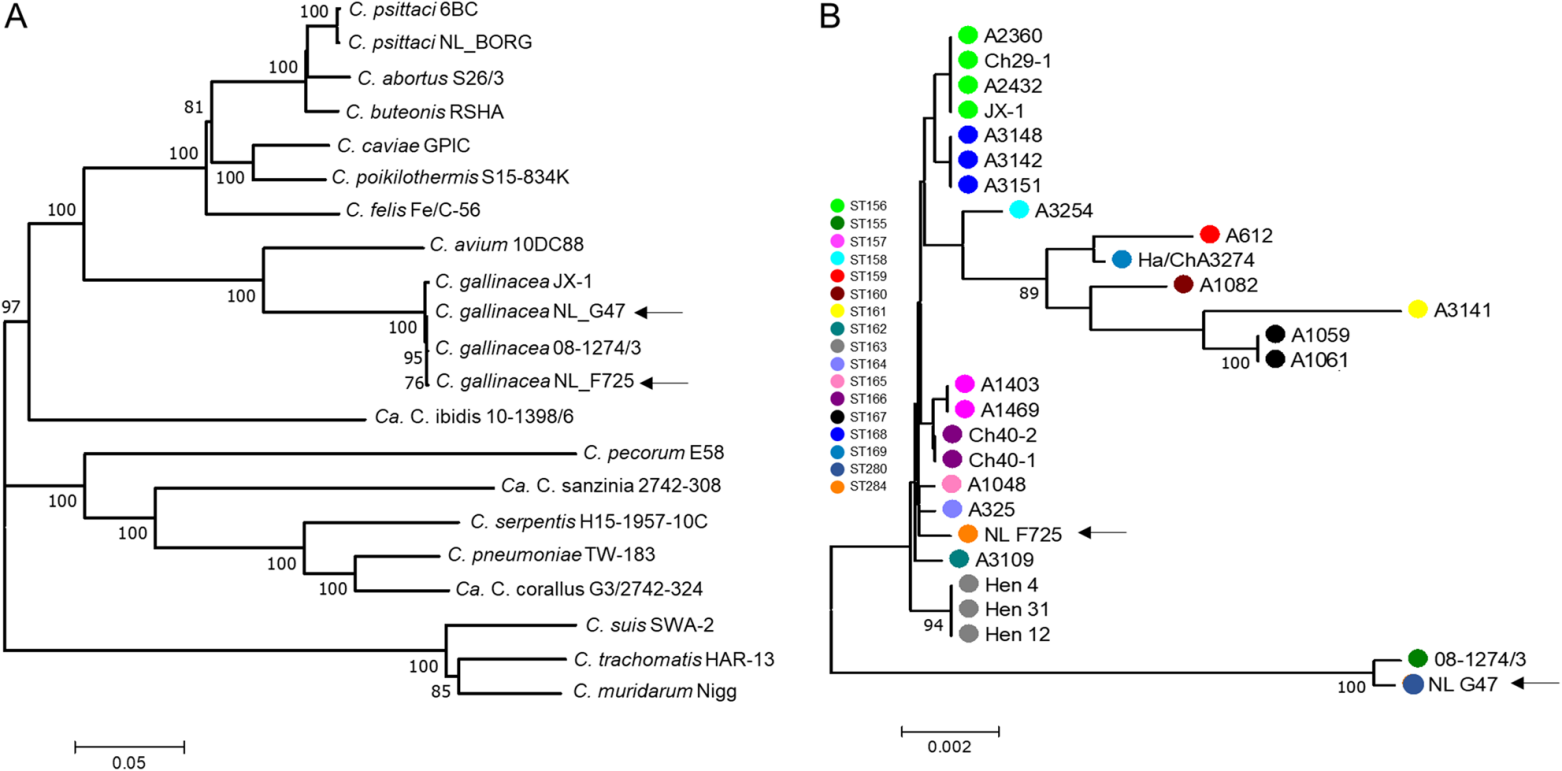
Phylogenetic analyses of concatenated sequences of *Chlamydia*. Concatenated sequences were aligned and analysed in MEGA7^37^. Numbers on tree nodes indicate bootstrap values over 75% of the main branches. Horizontal lines are scale for nucleotide substitutions per site. (**A**) Neighbor-Joining tree of concatenated sequences of 52 ribosomal genes (rMLST)^14^ of *Chlamydia* Type strains as well as three Candidatus species (*Ca.* C. corallus, *Ca.* C. ibidis and *Ca.* C. sanzinia), *C. psittaci* strain NL_Borg and two additional *C. gallinacea* strains. All *C. gallinacea* strains (Dutch strains indicated by an arrow) clustered together in a well-supported and distinct clade with *Chlamydia avium* as the closest relative. (**B**) Neighbor-Joining tree of concatenated sequences of 7 housekeeping genes fragments (MLST)^41^ of 27 *C. gallinacea* strains. Shared Sequence types (ST) in clades are indicated by color and STs are denoted by the color key.

After isolation in eggs and one passage in BGM cells, DNA of both isolates was sequenced to confirm their genetic identity. The genomes of NL_G47 and NL_F725 have a total length of 1,066,007 and 1,064,097 bp, respectively, and include the ~ 1.059 Mbp chromosome and a 7.5 kbp chlamydial plasmid (Table 1). Ribosomal MLST (rMLST)^14^ and phylogenetic analysis of concatenated rRNA genes confirmed that both isolates belong to *C. gallinacea* (Fig. 3A), whilst the MLST showed that both isolates are genetically diverse and assigned to unique sequence types (ST280 and ST284). Phylogenetically, these clustered in distinct clades, with NL_G47 forming a well-supported clade with the French isolate 08-1274/3, whilst NL_F725 clustered in a genetically diverse clade consisting of Chinese *C. gallinacea* strains (Fig. 3B).

**Table 1:** Genome descriptions of *C. gallinacea* NL_G47, *C. gallinacea* NL_F725 and *C. psittaci* NL_Borg.

	*C. gallinacea* NL_G47	*C. gallinacea* NL_F725	*C. psittaci* NL_Borg
Host	Chicken (*Gallus gallus*)	Chicken (*Gallus gallus*)	In-house reference strain
Anatomical site	Caecum	Caecum	Unknown
Clinical presentation	Asymptomatic	Asymptomatic	Unknown
Total No. of Illumina reads	1,912,918	1,762,101	3,029,302
Percent of mapped reads	72.92%	73.06%	93.46%
No. of de novo contigs^a^	12	19	11
N50	114,660	96,259	254,182
Average coverage depth	366X	181X	633X
%GC of de novo contigs	37.89%	37.89%	38.92%
Number of bp mapped against reference genome chromosome (% complete compared to reference strains^b^)	1,058,515 bp (99.89%)	1,057,023 bp (99.75%)	1,144,332 bp (98.5%)
Number of bp mapped against the reference plasmid	7492 bp	7492 bp	7552 bp
Number of predicted CDS	916	919	989
% Average nucleotide identity^c^	99.63% (SD: 1.09%) to *C. gallinacea* 08_1274/3	99.50% (SD: 1.53%) to *C. gallinacea* 08_1274/3	–
	99.42% (SD: 1.43%) to *C. gallinacea* JX-1	99.52% (SD: 1.31%) to *C. gallinacea* JX-1	–
	–	–	99.99% (SD: 0.04%) to *C. psittaci* NJ1
No of SNPs to reference strains^d^	2608 to *C. gallinacea* 08_1274/3	3328 to *C. gallinacea* 08_1274/3	65 to *C. psittaci* NJ1
Plasticity Zone length	15861 bp	15845 bp	29000 bp
Accession numbers	JAEMHG000000000	JAEMHH000000000	–

^a^*de novo* chlamydial contigs.

^b^Quast analyses using Short read assemblies where NL_G47 and NL_F725 were compared to 08_1274/3, and NL_Borg with NJ1.

^c^Average nucleotide identity (ANI) determination was performed at enve-omics.ce.gatech.edu/ani/ (Goris et al.^42^) using both best hits (one-way ANI) and reciprocal best hits (two-way ANI) between two genomic datasets with *C. gallinacea* 08_1274/3, *C. gallinaceae* JX-1 or *C. psittaci* NJ1 as the reference genome.

^d^SNPs identified using Snippy v4.6.0.

### Comparative genome analysis of *C. gallinacea* and *C. psittaci*

**Figure 4:**
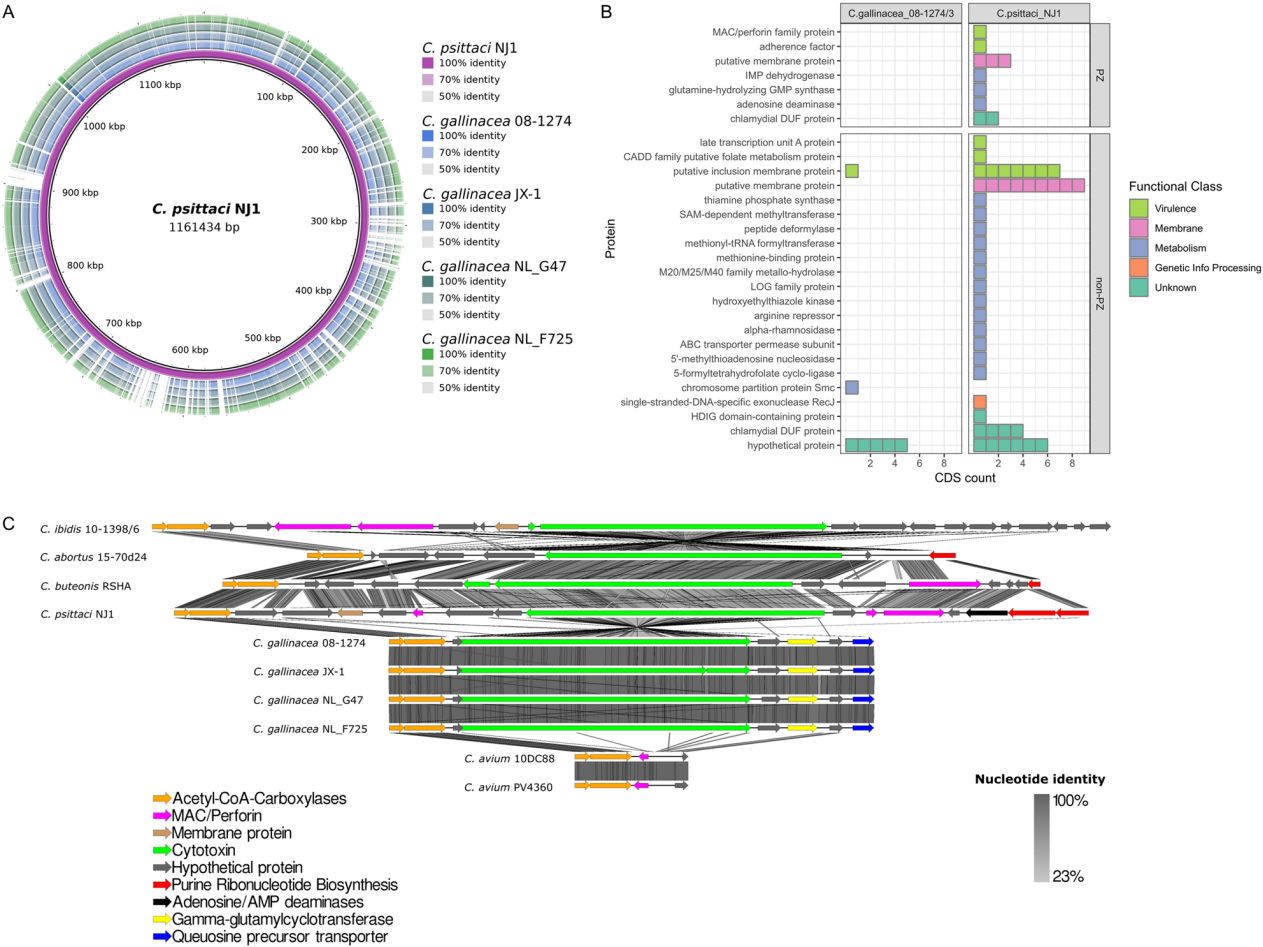
Genome comparison of *C. gallinacea* and *C. psittaci.* (**A**) Whole genome BLAST comparison between *C. psittaci* NJ1 and four *C. gallinacea* genomes (including Type strain 08-1274/03). The image is created with BLAST Ring Image Generator (BRIG)^49^ and the first ring corresponds to the genome that was used for the comparison. (**B**) CDS for which no homologue (alignment E score higher than 1 × 10^−3^) could be identified in *C. gallinacea* 08-1274/03 or *C. psittaci* NJ1. Every colored block in the figure corresponds to a CDS. The different proteins are categorized and colored according to their function and location. The figure was created using the *tidyverse* package and R v3.6.1^52,53^. (**C**) Graphical representation of the gene content of the PZs of representative *Chlamydia* species of avian origin including the Dutch *C. gallinacea* strains. Arrows represent PZ genes colored according to function (see key). Grey shading scale denotes % nucleotide identity. The image was created with Easyfig^50^.

To investigate genomic differences that might be related to the observed differences in the degree of pathology and mortality in eggs, the *C. gallinacea* and *C. psittaci* genomes were analysed and compared. As evaluated by whole genome alignments, *C. gallinacea* genomes NL_G47 and NL_F725 are syntenic with the same gene number and order, sharing at least 99.4% sequence identity with *C. gallinacea* strain 08-1274/3 (type strain; accession number NZ_CP015840.1) and JX-1 (accession number CP019792). All *C. gallinacea* genomes contain conserved hallmark chlamydial virulence genes coding for Incs, Pmps, T3SS and a Plasticity Zone (PZ) with a gene coding for the large cytotoxin (*toxB*) (Fig. 4A,C, Supplementary Fig. S3). Most sequence variation was found in several distinct chromosomal regions, namely in genes encoding the membrane proteins (e.g. *ompA* and *pmp*s), a conserved hypothetical protein, a phage tail protein, heme (*hemE*, and *hemN*) and glycogen (*glgP*) metabolism genes (Supplementary Data S2). The PZ, a region of high genetic variability in chlamydial species, was conserved in number of genes and sequence among the four *C. gallinacea* genomes with 99.3–99.8% nucleotide identity, but varied in gene content, namely lack of hypothetical protein, MAC/Perforin (MAC/P) and nucleotide metabolism genes, compared to the related avian species (Fig. 4C) Although, the length of the PZ of *C. gallinacea* is reduced compared to *C. psittaci*, it does contain an intact CDS for the cytotoxin (*toxB*), in contrast to the PZ of *C. avium* that lacks this gene. As observed previously, this locus has a premature stop codon in JX-1 strain (Fig. 4C).

The genome sequence of our in-house reference strain *C. psittaci* NL_Borg was almost identical (99.99% sequence identity) to reference strain *C. psittaci* NJ1 (accession number CP003798.1) with only 65 synonymous Single Nucleotide Polymorphisms (SNPs), evenly distributed across the chromosome. In the whole genome alignment, it was observed that the *C. psittaci* genome is 101.85 Kbp longer than the genome of *C. gallinacea* and contains 73 more CDSs (Fig. 4A).

Given that our newly sequenced genomes are syntenic and almost identical to the comparator reference genomes, but only cover 98.51–99.75% of the reference chromosome lengths (Table 1), genomes of the type strains 08-1274/3 and NJ1 were used as representatives for *C. gallinacea* and *C. psittaci* species, respectively, in a translated coding sequences (CDSs) comparison. With a local alignment approach, all translated CDSs of *C. gallinacea* 08-1274/3 (n = 913) and *C. psittaci* NJ1 (n = 986) were compared to each other to identify unique and/or highly variable regions (Supplementary Data S3). The plasmids of *C. gallinacea* and *C. psittaci* were not included, because they are syntenic with both eight CDSs encoding the conserved chlamydial plasmid proteins.

As expected in closely related species and analysed by both amino acid and sequence similarity analyses, the majority of CDSs have orthologues in both species. In *C. gallinacea,* for only seven CDSs an orthologue could not be identified in *C. psittaci* (Fig. 4B, Supplementary Data S4). Of those, one belonged to the family of putative Incs, a second had a metabolic function related to chromosome partition and the remaining five were hypothetical proteins with unknown function. Fifty-three CDS were unique to *C. psittaci* relative to *C. gallinacea* (Supplementary Data S4). Ten of these CDSs were located at the PZ coding for proteins such as the Membrane Attack Complex/Perforin domain-containing protein (MAC/PF), proteins involved in purine metabolism (*gua*AB-ADA operon), adherence domain and a putative membrane protein.

Outside the PZ, 18 of the unique CDS of *C. psittaci* were related to previously characterised potential virulence factors (Fig. 4B, Supplementary Data S4). Most of these proteins belonged to the family of putative Inc proteins, membrane proteins and conserved hypothetical proteins. The remaining unique CDS were related to metabolism or to CDS coding for proteins of unknown function. Additional analysis of secretion signals of T3SS effector CDSs, important in *Chlamydia* virulence, revealed that a serine protease referred to as chlamydial protease-like activating factor (CPAF) is not predicted to be secreted in *C. gallinacea* in contrast to *C. psittaci*^15^. However, *C. psittaci* orthologues of the recently described T3SS that associate with the host’s inner nuclear membrane (SINC), and translocated actin-recruiting phosphoprotein (TARP) were identified and predicted to be secreted (Supplementary Data S5).

Overall, the analysis revealed the novel *C. gallinacea* genomes NL_G47 and NL_F725 have at least 99.5% sequence identity to the Type strain 08-1274/3 and include the hallmark chlamydial virulence genes. However, *C. psittaci* has a larger set of genes that are related to virulence and metabolism, including more *inc*s, *pmp*s, T3SS effectors and additional genes in the PZ.

## Discussion

In this study, the pathogenic potential of two new chicken-derived *C. gallinacea* strains (NL_G47 and NL_F725) were investigated combining classical in vitro methods using embryonated chicken eggs and whole-genome analyses. During isolation of NL_G47 and NL_F725, pathogenic changes were observed that also have been described for other *Chlamydia* species^16^, such as deep red colour (rubor), cyanotic toes and skin haemorrhage of the embryo. Mortality in embryonated eggs after yolk sac inoculation with *C. gallinacea* has been reported by Guo et al.^4^, but was not mentioned by Laroucau et al.^9^.

The layer flocks from which the strains originated were apparently healthy, which is in line with observations from other field studies^4,9,10^. It could not be evaluated if *C. gallinacea* infection led to impaired production as data on egg production were not collected in this teaching flock. The duration and frequency of shedding during *C. gallinacea* infection was only assessed to a limited extent due to the sampling strategy.

In the flock of strain NL_F725, the *C. gallinacea* infection preceded an infection with Infectious Laryngotracheitis (ILT) resulting in preventative culling to limit the spread of ILT. Whether a primary infection of *C. gallinacea* enhances infection with other pathogens or whether co-infection might exacerbate the disease outcome, is currently unknown. For *C. gallinacea*, only co-infections with *C. psittaci* have been reported in chickens without details about the clinical outcome^5,17^. For *C. psittaci*, it has been suggested that co-infections with respiratory pathogens might lead to a more severe disease outcome^18,19^. The effect of co-infection could be a topic for future investigations.

In titration experiments in embryonated eggs, the pathogenicity of *C. gallinacea* was compared to a virulent *C. psittaci* poultry strain. The infectious dose and mortality in *C. gallinacea* infected eggs was lower compared to *C. psittaci* infected eggs. Furthermore, although the observations were limited, a small difference in pathogenicity between both *C. gallinacea* strains was observed. *C. gallinacea* NL_G47 infection resulted in mortality up to the 10^−2^ dilution (1 of 5 eggs), while no mortality was observed in the 10^−2^ dilution with strain NL_F725 (0 of 3 eggs). As follow, this is a first indication of a possible difference in pathogenicity between genetically different *C. gallinacae* strains, but needs to be confirmed due the low number of observations.

Furthermore, a higher mortality in *C. psittaci* infected eggs compared to *C. gallinacea* is in line with findings in available field and experimental studies. In these studies, *C. gallinacea* infection led to reduced weight gain in chickens and the absence of clinical symptoms, while exposure to a known high virulent *C. psittaci* strain can lead to severe systemic infections in chickens and turkeys^4,10,20,21^. In contrast, exposure to a less virulent *C. psittaci* strain resulted in mild respiratory symptoms indicating the importance of detailed strain knowledge and infection conditions^20^.

The difference in infectious dose and mortality between *C. gallinacea* and *C. psittaci* in embryonated eggs might be a result of a shorter development cycle of *C. psittaci*. The development cycle of *C. gallinacea* takes about 60 to 72 h while that of *C. psittaci* about 50 h^3,22^. In the experiments, all eggs were harvested at the same time point, which could mean *C. psittaci* was able to replicate to a higher number of bacteria. The difference in replication time could therefore contribute to the virulence of *C. psittaci*.

To get further insight into the genetic background of *C. gallinacea* in relation to pathogenicity, additional genomic comparisons were performed. Both *C. gallinacea* isolates were at least 99.4% identical to *C. gallinacea* Type strain 08-1274/3, with genetic diversity contained to several distinct chromosomal regions, and had a smaller set of potential virulence genes compared to *C. psittaci*. However, the question remains if a smaller set of virulence genes is a disadvantage for the particular isolate or species involved and determines the observed difference in pathogenicity. The closest genetic relative of *C. gallinacea*, *C. avium*, also has a reduced set of virulence genes compared to *C. psittaci*, and exhibits the smallest PZ region of all *Chlamydia*, but in cases involving pigeons and psittacines infection does lead to clinical signs and mortality^23,24^.

Moreover, *C. gallinacea* does contain all hallmark virulence factors such as Incs, Pmps T3SS and an intact cytotoxin in the PZ, except in strain JX-1 ^12^. In addition, *C. gallinacea* has genes encoding the well-known T3SS effectors TARP and SINC that play a role in the pathogenesis of *Chlamydia* spp. In *C. psittaci*, TARP influences the active uptake in the host cell and SINC targets the nuclear envelope where it is hypothesized to interact with host proteins that control nuclear structure, signalling, chromatin organization, and gene silencing^25,26^. Future studies need to confirm if both effectors are indeed secreted in *C. gallinacea,* with which host proteins they interact, and whether differences in gene expression can be identified that might play a role in pathogenicity.

Based on our current results in embryonated eggs and the genomic comparisons, it is too early to conclude that *C. gallinacea* is a phenotypical commensal. Although less pathogenic than the *C. psittaci* strains of avian origin, *C. gallinacea* does possess the hallmark *Chlamydia* virulence genes, and infection does lead to mortality in embryonated chicken eggs after yolk sac inoculation. Furthermore, there might be small differences in virulence between *C. gallinacea* strains. Additional pathogenesis studies in chickens, including predisposing conditions such as co-infections, are therefore needed to further elucidate the pathogenic potential of *C. gallinacea* and possible strain differences. These future studies will help to assess the importance of this pathogen for poultry industry.

## Methods

### Ethical statement and biosafety

The cloacal and caecal sampling of the chickens was approved by the Dutch Central Authority for Scientific Procedures on Animals and the Animal Experiments Committee (permit number AVD108002016642) of Utrecht University (the Netherlands). All procedures were conducted in accordance with national regulations on animal experimentation and in compliance with the ARRIVE guidelines^27^ where applicable. No ethical approval is required for work with embryonated chicken eggs until day 18 according to Dutch Law.

All culture work with *C. gallinacea* was performed under biosafety level 2 and all culture work with *C. psittaci* under biosafety level 3.

### Sample collection, inoculum preparation and isolation of *Chlamydia*

#### Sample collection and inoculum preparation

Layer flocks at the Faculty of Veterinary Medicine in Utrecht, the Netherlands were monitored for the presence of *C. gallinacea* with boot sock sampling. The flocks were obtained from commercial laying hen rearing farms at 18-weeks of age and had an average size of 50 hens that were distributed evenly over two pens. Background data on the flock are supplied in Supplementary Fig. S1 and Supplementary Data S1. From each pen, environmental boot sock samples (Poultry Boot Swabs, BioTrading) were collected monthly. After collection, the boot socks were suspended in 100 ml Dulbecco’s Phosphate Buffered Saline (DPBS, Gibco, Life Technologies Limited). The suspension was centrifuged 15 min at 500 × *g* and 500 µl of the supernatant was used for DNA isolation. When the boot socks were PCR positive for *Chlamydia*, individual cloacal swabs and caeca were collected. Cloacal swabs were stored in one millilitre Sucrose Phosphate Glutamate (SPG) and caeca in ten percent weight per volume (w/v) according to standard protocols^28,29^. SPG contains sucrose (75 g/litre), KH_2_PO_4_ (0.52 g/litre), K_2_HPO_4_ (1.25 g/litre) and L-glutamic acid (0.92 g/litre). Before use, fetal bovine serum (0.1 ml/ml), amphotericin B (4 µg/ml), gentamicin (40 µg/ml and vancomycin (25 µg/ml) were added. Samples were stored at − 80 °C.

To prepare the inoculum for the eggs, swabs were thawed at room temperature for approximately one hour. Swabs were centrifuged for ten minutes at 500 × *g* and 200 µl of the supernatant was used for inoculation. Caeca were prepared following two methods. For the isolation of NL_G47 the caecum was cut lengthways in parts of approximately two cm. Subsequently the parts were washed in SPG and the epithelium was removed by scraping with a scalpel. The scrapings of epithelium were washed in two ml of SPG and the suspension was filtered over a 0.8 µm filter (Acrodisc Syringe Filter, Pall Life Sciences). After one hr of incubation at room temperature the suspension was used for inoculation. For the isolation of NL_F725, caeca were homogenized in a ten percent w/v suspension in an ULTRA-TURRAX tube (BMT-20-S, IKA) on an ULTRA-TURRAX Tube Drive (IKA) at 6000 RPM for 90 s and switching direction every 30 s. The suspension was centrifuged at 500 × *g* for 15 min and the supernatant was used for culturing as described below.

#### Inoculation of embryonated SPF chicken eggs

Specific-pathogen-free (SPF) embryonated chicken eggs were delivered after five days of incubation, candled to check viability and incubated overnight at 37.5–38 °C and 65% relative humidity in small egg incubators (Octagon 20 Advance, Brinsea). Inoculation was performed at day six of incubation (one day after delivery).

Before inoculation, the eggs were candled, and the air chamber was marked with a pencil. The eggs were cleaned with a wipe drenched in 70% ethanol. In the middle of the area of the marked air chamber, a hole was drilled with a 0.8 mm engraving bit (26150105JA, Dremel). Subsequently, the eggs were moved to a flow cabinet and sprayed with 70% ethanol. Per egg, 200 µl was inoculated in the yolk sac with a one millilitre syringe and a 22G × 40 mm needle. The full needle was inserted perpendicularly into the drilled hole.

Per clinical sample, four eggs were inoculated. As a negative control, two eggs were inoculated with DPBS (Gibco, Life Technologies Limited) and, as a positive control, two eggs were inoculated with *C. gallinacea* strain 08DC65. Strain 08DC65 was obtained from the Friedrich Loeffler Institute in Jena, Germany.

After inoculation eggs were wiped with 70% ethanol and the hole was closed with a droplet of nail polish. The eggs were placed in the egg incubators and incubated until day 16 or until mortality. At day 16, eggs were chilled overnight at 4 °C to euthanise the embryo non-invasively.

#### Candling of embryonated SPF chicken eggs

Mortality was monitored by daily candling. With candling, the appearance of vessels and movement of the embryo was monitored^30^. The result of candling was graded:

- no abnormalities observed: vessels are visible, movement of the embryo
- abnormalities observed: congestion or bleeding from vessels, decreased movement of the embryo
- mortality: no or less vessels visible and no movement of the embryo.

When abnormalities were observed an extra candling was performed on the same day. After mortality or an increase in the severity of the abnormalities, eggs were chilled overnight at 4 °C until harvesting.

#### Harvesting of embryonated SPF chicken eggs

Mortality within three days after inoculation (day nine of incubation) was considered as acute mortality inconsistent with a *Chlamydia* infection^16^. These eggs were disinfected with 70% ethanol, opened at the air sac side and checked for any visual deformations. Furthermore, a sheep blood agar plate was inoculated with a loopful from the yolk sac and incubated overnight at 37 °C to check for bacterial contamination.

Eggs were harvested for the isolation of *C. gallinacea* when mortality occurred from day nine of incubation or when no mortality was observed at day 16 of incubation. At harvesting the part of the egg shell covering the air sac was removed, and subsequently the egg shell membrane and the allantois membrane were opened with disposable tweezers. The allantoic fluid was removed with a pipette, the egg was then emptied in a Petri dish to harvest the yolk sac membrane. The yolk sac membrane was weighed and transferred to an ULTRA-TURRAX tube (BMT-20-S, IKA). Depending on the volume of the yolk sac and the size of the tube, SPG buffer was added and the yolk sac membrane was homogenized on an ULTRA-TURRAX Tube Drive (IKA) for 90 s (switching between forward and reverse every 30 s) at 6000 RPM. The suspension was transferred to 50 ml Falcon tubes and SPG buffer was added until a 20% w/v suspension.

The yolk sac membranes from eggs inoculated with the same sample and harvested at the same day, were pooled to create one homogenous batch of an isolate. A 10 µl droplet of the yolk sac suspension was spotted in duplo on glass slides and air dried. The glass slides were tested with the IMAGEN *Chlamydia* test kit (immunofluorescence test, IFT) according to manufacturer’s instructions (Thermo Scientific). Two hundred µl of the suspension was used for PCR testing.

#### Isolation in cell culture

Isolation and propagation in cell culture was performed as described earlier^23^. Briefly, Buffalo Green Monkey (BGM) cells were seeded with Dulbecco’s Modified Eagle Medium (DMEM, Gibco, Life Technologies Limited) and 10% serum in 24-well plates (Greiner Bio-One GmbH, Germany). The plates were incubated at 37 °C with 5% CO_2_ in a humidified incubator until 80% confluency of the monolayer. After inoculation, the plates were centrifuged at 2450 × g and 37 °C for 60 min and subsequently incubated for two hours. The medium was then replaced with UltraMDCK serum-free medium (Lonza). At day one and day four, 200 µl of the supernatant was collected for PCR to monitor replication. Plates were harvested at day four for DNA isolation, further passaging or storage at – 80 °C.

### Titration experiments in embryonated SPF chicken eggs

The isolated *C. gallinacea* strains NL_G47 and NL_F725, and *C. psittaci* strain NL_Borg were tested in titration experiments. Strain NL_Borg was selected because it is genetically closely related to strain FalTex and NJ1, which are both isolated from outbreaks in poultry (turkeys)^31^.

To standardise the inocula before the start of the titration experiments, all three strains were passaged three times in embryonated eggs under similar conditions. The third passage yolk sac membrane suspensions were used to prepare tenfold serial dilutions in DPBS (Gibco, Life Technologies Limited) for inoculation of the yolk sac of six-day incubated chicken eggs. The eggs were incubated at 37 °C and 65% relative humidity in egg incubators (Octagon 20 Advance, Brinsea). After mortality or six days after inoculation the eggs were chilled overnight at 4 °C and harvested as described earlier.

In a first experiment the range for the dilution series was defined by inoculating a limited number of eggs per dilution. In a subsequent experiment the range was limited to four dilution steps. Per dilution step, four or five eggs were inoculated with 200 µl suspension. Two or more eggs were inoculated with sterile DPBS (Gibco, Life Technologies Limited) as a negative control and, as a positive control, two eggs were inoculated with a lower dilution than the range that was used in the experiment.

After each titration experiment the 50% egg infective dose (EID_50_) and, when possible, the 50% egg lethal dose (LD_50_) per ml inoculum was calculated according the Spearman–Karber method^32,33^. The difference in EID_50_ between strains was assessed using the Wilcoxon–Mann–Whitney test.

### Histology and immunohistochemistry

From infected and non-infected eggs, the chorioallantoic membrane, yolk sac and embryo were harvested for histology and immunohistochemistry. After fixation in 10% neutral buffered formalin, tissues were routinely processed into paraffin blocks. Four µm sections were cut and collected on coated glass slides. Sections were stained with haematoxylin–eosin (HE) or immuno-stained with a polyclonal anti-*Chlamydia* antibody (LS-C85741) and a monoclonal anti-*Chlamydia* antibody (MBS830551).

For the polyclonal antibody the antigen was retrieved by proteolysis-induced epitope retrieval (0.1% Trypsin in TBS for 30 min at 37 °C). For the monoclonal antibody heat-induced epitope retrieval was used (citrate buffer, pH 6.0, 21 °C for five min). The primary antibody (dilution 1:100) was incubated for 60 min. HRP EnVision anti-Mouse or HRP Envision anti-Rabbit (Dakopatts) were used as a secondary antibody for 30 min, depending on the nature of the first antibody. Subsequently, sections were incubated for five min in DAB + substrate (Dakopatts) and then counterstained with Mayer’s haematoxylin.

### DNA extraction, PCR and genome sequencing

Five hundred µl of the sample suspensions, washing suspension, yolk sac suspension or cell culture supernatant was used for DNA extraction. DNA extraction was performed with a MagNA Pure LC total Nucleic Acid Isolation kit in the MagNA Pure system (Roche Diagnostics, Almere, the Netherlands). Samples were tested with a *Chlamydiaceae* PCR targeting the 23S rRNA and *C. gallinacea* PCR targeting the *enoA* gene or *C. psittaci* PCR targeting the *ompA* gene as described earlier^10,34^.

For genome sequencing, twenty-four-well cell culture plates were freeze-thawed twice and the cells were subsequently harvested for DNA extraction as described earlier^23^. DNA was isolated according to the DNeasy Blood and Tissue kit (Qiagen GmbH, Germany).

The DNA samples were prepared for Illumina sequencing using the SMARTer ThruPLEX DNA-Seq kit (Takara Bio, USA) according to manufacturer protocol. Quality control of the library preparation was performed on a Tapestation 2200 (Agilent Technologies, Germany) and the DNA concentration was determined on a Clariostar (BMG Labtech, the Netherlands) with use of the Quant-IT PicoGreen dsDNA kit (Invitrogen Ltd, UK). Sequencing was performed on an Illumina MiSeq platform. The complete genome and plasmid sequences were assembled using SKESA 2.4.0^35^. Contigs containing sequences of BGM cells were removed prior to subsequent analysis.

Assembled contigs (from Illumina short reads) were annotated using the PGAP pipeline using *C. gallinacea* Type Strain 08-1274/3 (accession number NZ_CP015840.1) as the reference genome for the newly isolated *C. gallinacea* strains and *C. psittaci* NJ1 (accesion number CP003798.1) for *C. psittaci* NL_Borg^36^. All data are available in the NCBI database under BioProject number PRJNA687129 (including reads available under SRR15184193; SRR15184194 and SRR15212117)and the publicly available Bacterial Isolate Genome Sequence Database (BIGSdb) ((http://pubmlst.org/chlamydiales) (*C. gallinacea* isolates NL_G47 (id: 4548) and NL_725 (id: 4560) and *C. psittaci* NL_Borg (id: 4561)).

### Molecular typing and phylogenetic analysis

Sequence types for our strains were determined using contigs deposited and queried against the *Chlamydiales* PubMLST database (http://pubmlst.org/chlamydiales). Phylogenetic trees were generated by exporting gene sequences from the *Chlamydiales* database (http://pubmlst.org/chlamydiales) as an XMFA file containing each locus as an aligned block. The XMFA file was converted to an aligned concatenated sequence for Neighbor-Joining tree analysis using the Maximum Composite Likelihood model in MEGA7^37^. Bootstrap tests were for 1000 repetitions^38–40^.

For rMLST, complete sequences (~ 22.000 bp) of 52 genes encoding ribosomal proteins (*rps*) were analysed^14^. The *rps* gene *rpmD*, encoding the 50S ribosomal protein L30 is absent in genomes of *Chlamydia* isolates analysed so far. For MLST, sequences of fragments (400–500 base pairs) from seven housekeeping genes (*enoA*, *fumC*, *gatA*, *gidA*, *hemN*, *hlfX*, *oppA*) were analysed^41^. Isolates used for rMLST and MLST including provenance and allelic profile data are listed in Supplementary Data S6.

### Comparative genome analyses

Average nucleotide identity (ANI) determination for the newly sequenced *C. gallinacea* genomes was performed using the ANI calculator available at enve-omics.ce.gatech.edu/ani/^42^, whilst the genome completeness based on the percent of bases aligned to the reference genome and quality of the assemblies was estimated using Quast^42–44^. SNPs in contigs assembled from Illumina reads, were identified using Snippy v4.6.0^45^.

*C. gallinacea* pairwise genome comparisons were performed using the Geneious Prime 2020.2 platform (https://www.geneious.com). Our strains were compared against *C. gallinacea* strain 08-1274/3 (accession number NZ_CP015840.1) and JX-1 (accession number CP019792). The genomic regions of interest and/or polymorphic loci were extracted from the analysed genomes and aligned with MAFFT and/or Clustal Omega (as implemented in Geneious Prime) for further nucleotide and/or translated protein sequence analyses performed using DNASp 6.0^46^. The total number of polymorphisms (and gaps), % nucleotide and amino acid sequence identity, number of haplotypes and haplotype diversity (Hd), and ratios of the rates of non-synonymous to synonymous nucleotide substitutions per site (dn/ds) averaged over the entire gene alignment were calculated.

As the Type 3 Secretion System (T3SS) play a key role in the interaction of *Chlamydia* and hosts, EffectiveDB (http://effectivedb.org) was used to predict the T3S secreted proteins of *C. gallinacea*. For prediction the standard Effective T3 classification module 2.0.1 was used with a cut-off score of 0.9999^47^. Similarly, to predict transmembrane *C. gallinacea* proteins, and identify inclusion membrane proteins characterised by bilobed hydrophobic domains, TMHMM 2.0 server (https://services.healthtech.dtu.dk/service.php?TMHMM-2.0) was used^48^.

The visualisation of nucleotide BLAST comparisons of our newly sequenced draft *C. gallinacea* genomes to published *C. gallinacea* genomes 08-1274/3 and JX-1, and/or *C. psittaci* NJ1 (accession number CP003798.1) was performed with BLAST Ring Image Generator (BRIG)^49^. Visualisation of the BLAST comparison, sequence identity and genomic structure of the plasticity zone for *C. gallinacea* and those from other related species, was performed using EasyFig, with the -tblastx option with a minimum E-value of 1 × 10^−3^ used as BLAST parameters for EasyFig^50^.

For the identification of orthologous genes in *C. gallinacea* and *C. psittaci*, an all-vs.-all comparison of the translated coding sequences (CDSs) was performed using global sequence alignment of each CDS. Translated CDSs were aligned using DIAMOND v0.9.14 and the best hit for each query was selected^51^. Only hits with an expect (E) value less than 1 × 10^−3^ were included. CDS with no hits or hits with an E-value above the threshold were further investigated and the annotation artefacts were removed. The remaining CDS were assigned unique. In addition, all CDS were investigated using both nucleotide and translated amino acid sequence blast analyses. Results of the alignment were structured and visualized using the *tidyverse* package and R v3.6.1^52,53^.

## Supplementary Information

Supplementary Information 1.

Supplementary Information 2.

Supplementary Information 3.

Supplementary Information 4.

Supplementary Information 5.

Supplementary Information 6.

Supplementary Figure S1.

Supplementary Figure S2.

Supplementary Figure S3.

Supplementary Table S1.

Supplementary Legends.
