# Supplementary figures and images for: Genetic and phenotypic analysis of the pathogenic potential of two novel Chlamydia gallinacea strains compared to Chlamydia psittaci
Source: Sci Rep. 2021 Aug 13;11:16516. doi: 10.1038/s41598-021-95966-9 (PMC8363750; doi:10.1038/s41598-021-95966-9)

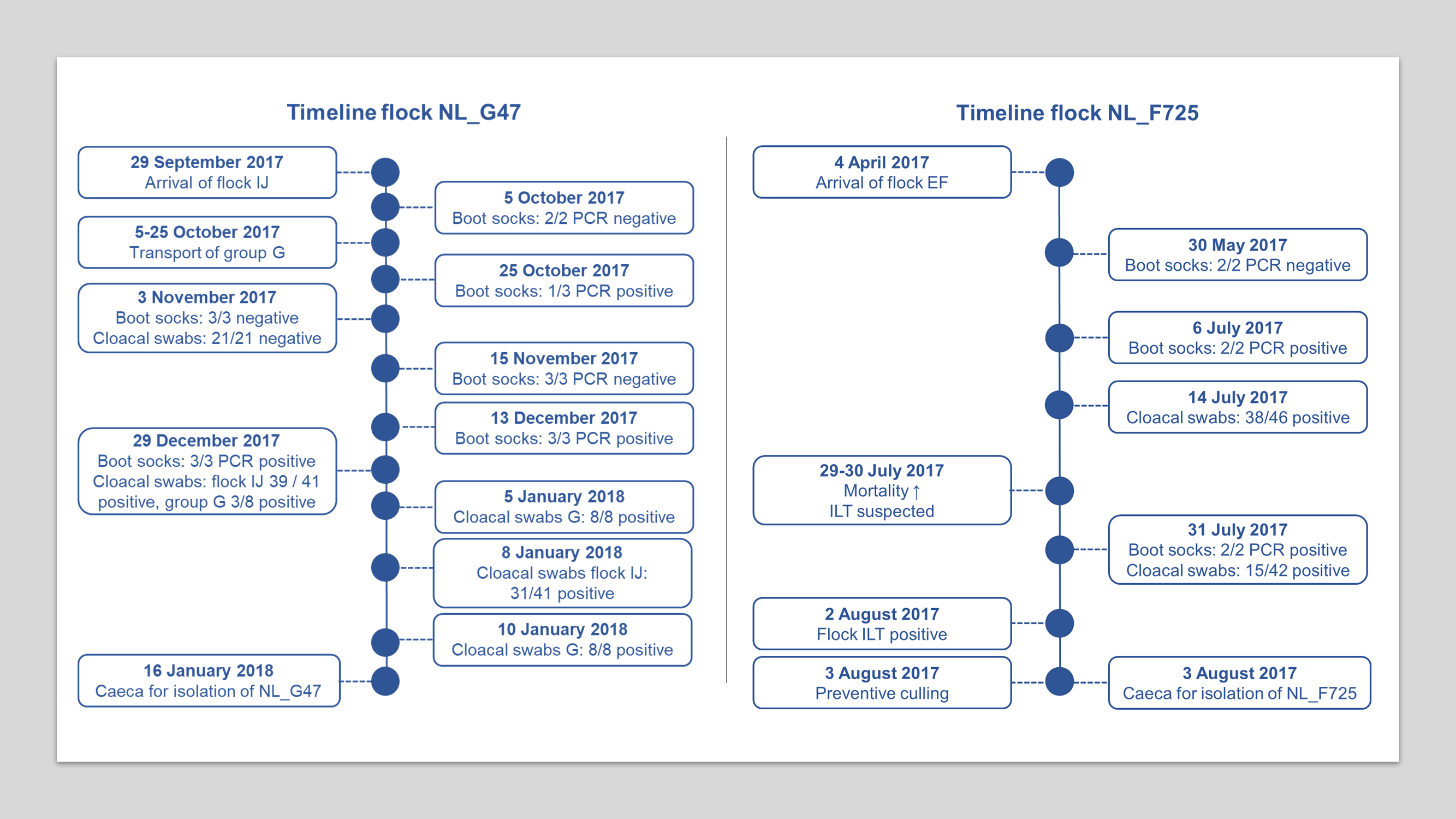

Supplement: Supplementary file 7 — Supplementary Figure S1. [file 41598_2021_95966_MOESM7_ESM.tif]

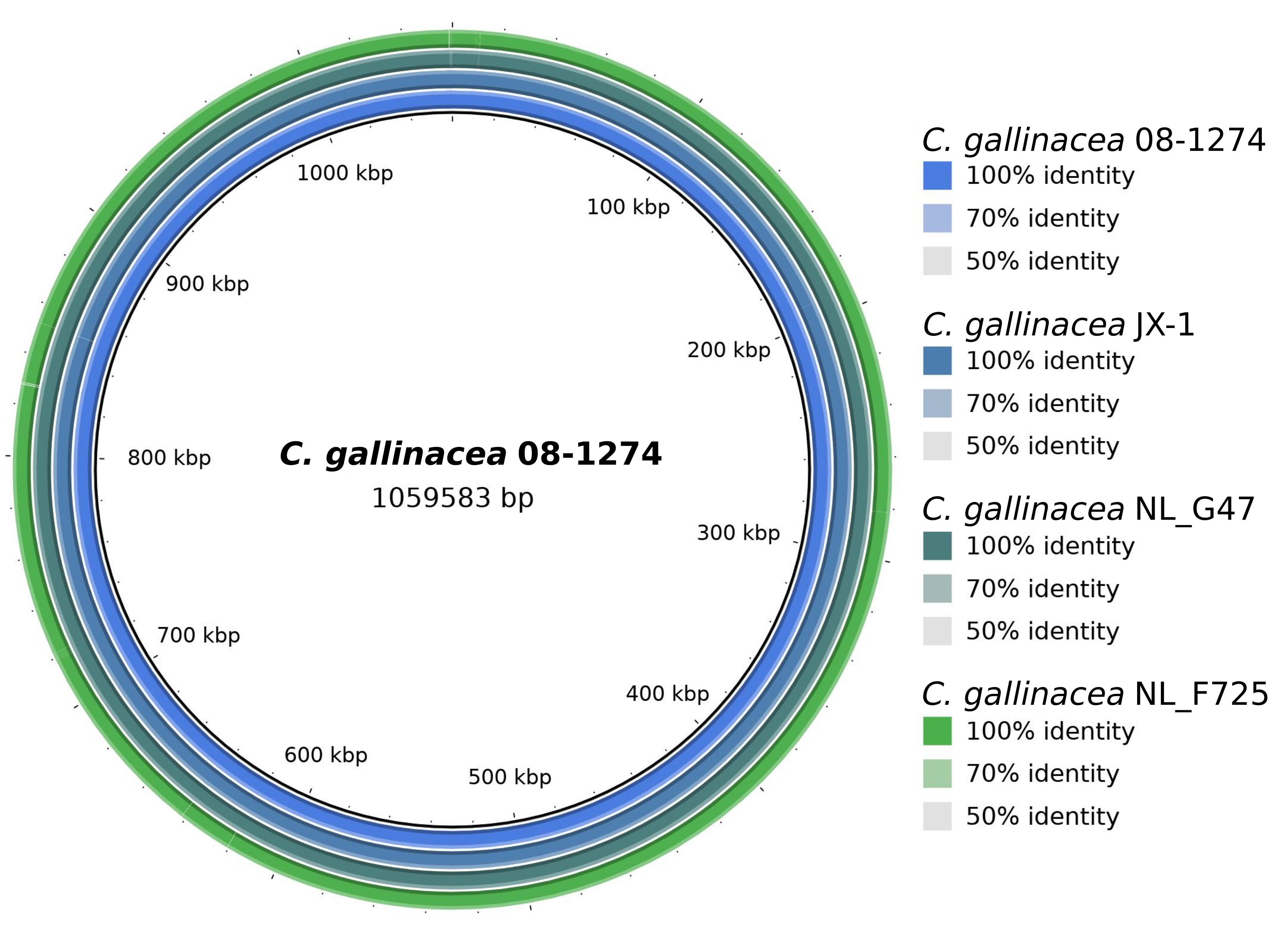

Supplement: Supplementary file 9 — Supplementary Figure S3. [file 41598_2021_95966_MOESM9_ESM.png]
