# Supplementary material for: Genetic and phenotypic analysis of the pathogenic potential of two novel Chlamydia gallinacea strains compared to Chlamydia psittaci
Source: Sci Rep. 2021 Aug 13;11:16516. doi: 10.1038/s41598-021-95966-9 (PMC8363750; doi:10.1038/s41598-021-95966-9)
Supplement: Supplementary file 8 — Supplementary Figure S2. [file 41598_2021_95966_MOESM8_ESM.docx]

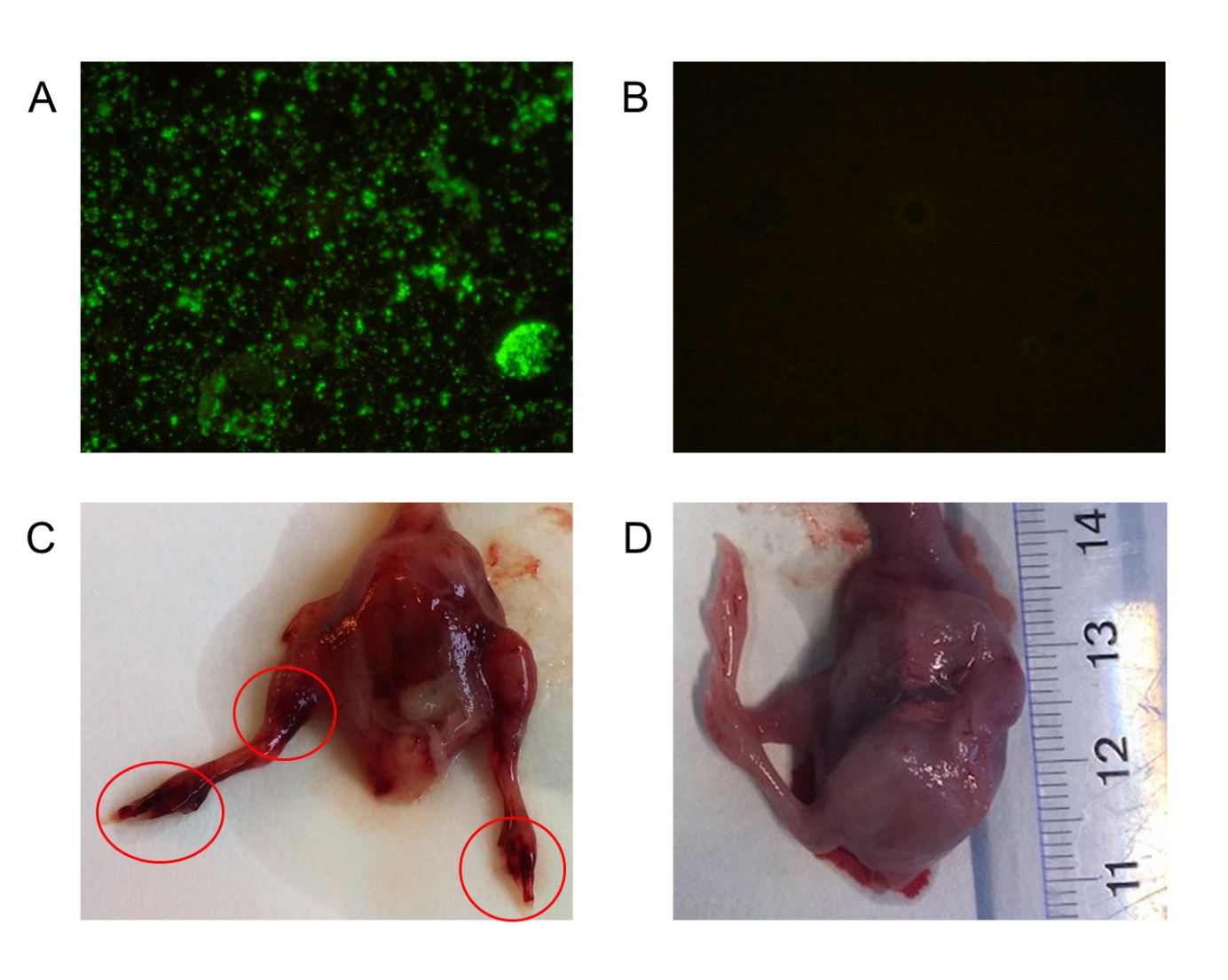


Fig S3. Results of yolk sac stamp stains and pathology in embryos during isolation in embryonated eggs.

(A) Positive and (B) negative immunofluorescence staining of yolk sac suspension stamps.

(C) Embryo of *Chlamydia gallinacea* infected egg and (B) an embryo of a negative control egg at day 8 after inoculation and day 14 of incubation. In the embryo of the infected egg (C) haemorrhages of the toes and upper leg are visible (red encircled). Furthermore the embryo of the infected egg is more red in color than the embryo of the uninfected egg.
