# Supplementary material for: Genetic and phenotypic analysis of the pathogenic potential of two novel Chlamydia gallinacea strains compared to Chlamydia psittaci
Source: Sci Rep. 2021 Aug 13;11:16516. doi: 10.1038/s41598-021-95966-9 (PMC8363750; doi:10.1038/s41598-021-95966-9)
Supplement: Supplementary file 10 — Supplementary Table S1. [file 41598_2021_95966_MOESM10_ESM.docx]

Table S4. Titration results of *C. gallinacea* NL_G47, *C. gallinacea* NL_F725 and *C. psittaci* NL_Borg

| **Results per isolate** | **Undiluted** | **1E+01** | **1E+02** | **1E+03** | **1E+04** | **1E+05** | **1E+06** | **1E+07** | **1E+08** | **1E+09** |
| --- | --- | --- | --- | --- | --- | --- | --- | --- | --- | --- |
| *C. gallinacea* NL_G47^*^ |  |  |  |  |  |  |  |  |  |  |
| IFT negative | 0 | 0 | 0 | 0 | 2 | 18 | 26 | 14 | nt | nt |
| IFT positive, no mortality | 2 | 1 | 4 | 23 | 28 | 12 | 4 | 0 | nt | nt |
| IFT positive and mortality | 5 | 2 | 1 | 0 | 0 | 0 | 0 | 0 | nt | nt |
| Total number of eggs | 7 | 3 | 5 | 23 | 30 | 30 | 30 | 14 | nt | nt |
| % IFT positive, no mortality | 29% | 33% | 80% | 100% | 93% | 40% | 13% | 0% | nt | nt |
| % IFT positive and mortality | 71% | 67% | 20% | 0% | 0% | 0% | 0% | 0% | nt | nt |
|  |  |  |  |  |  |  |  |  |  |  |
| *C. gallinacea* NL_F725 |  |  |  |  |  |  |  |  |  |  |
| IFT negative | nt | nt | 0 | 0 | 0 | 1 | 7 | 5 | nt | nt |
| IFT positive no mortality | nt | nt | 3 | 5 | 8 | 7 | 3 | 0 | nt | nt |
| IFT positive and mortality | nt | nt | 0 | 0 | 0 | 0 | 0 | 0 | nt | nt |
| Total number of eggs | nt | nt | 3 | 5 | 8 | 8 | 10 | 5 | nt | nt |
| % IFT positive, no mortality | nt | nt | 100% | 100% | 100% | 88% | 30% | 0% | nt | nt |
| % IFT positive and mortality | nt | nt | 0% | 0% | 0% | 0% | 0% | 0% | nt | nt |
|  |  |  |  |  |  |  |  |  |  |  |
| *C. psittaci* NL_Borg |  |  |  |  |  |  |  |  |  |  |
| IFT negative | nt | nt | 0 | nt | 0 | 0 | 0 | 2 | 7 | 5 |
| IFT positive no mortality | nt | nt | 0 | nt | 0 | 0 | 1 | 6 | 1 | 0 |
| IFT positive and mortality | nt | nt | 2 | nt | 6 | 10 | 14 | 6 | 0 | 0 |
| Total number of eggs | nt | nt | 2 | nt | 6 | 10 | 15 | 14 | 8 | 5 |
| % IFT positive, no mortality | nt | nt | 0% | nt | 0% | 0% | 7% | 43% | 13% | 0% |
| % IFT positive and mortality | nt | nt | 100% | nt | 100% | 100% | 93% | 43% | 0% | 0% |

nt = not tested; all isolates were passaged three times in eggs; the differences in number of eggs per dilution step are caused by the number of experiments, the dilution range used in every experiment, mortality before day three and the availability of surplus eggs; ^*^ For strain *C. gallinacea* G47 the titration results of two different batches are shown.
