# Supplementary material for: Genetic and phenotypic analysis of the pathogenic potential of two novel Chlamydia gallinacea strains compared to Chlamydia psittaci
Source: Sci Rep. 2021 Aug 13;11:16516. doi: 10.1038/s41598-021-95966-9 (PMC8363750; doi:10.1038/s41598-021-95966-9)
Supplement: Supplementary file 11 — Supplementary Legends. [file 41598_2021_95966_MOESM11_ESM.docx]

# Supplementary captions

## Supplementary captions figures

**Fig S1 . Timeline with flock information.** In the timeline sampling data are provided of the flocks from which *C. gallinacea* strain NL_G47 and NL_F725 could be isolated. All boot sock and cloacal samples were tested with the 23S *Chlamydiaceae* PCR. In the caecal samples for isolation the presence of *C. gallinacea* was confirmed with the *C. gallinacea* PCR. ILT positivity of flock NL_F725 was confirmed with an antibody ELISA.

**Fig S2. IFT of the yolk sac membrane and pathologic lesions of the embryo.** A and B show a positive and a negative IFT result of the yolk sac membrane. C and D show an embryo of an uninfected and a *C. gallinacea* infected egg with haemorrhages of the toes and upper leg.

**Fig S3 Genome comparison of four different *C. gallinacea* strains.** Whole genome BLAST comparisons between four *C. gallinacea* genomes created with BLAST Ring Image Generator (BRIG)^53^

## Supplementary captions tables

**Table S1. Results of titration experiments.** In the table the data from all the separate titration experiments were included.

## Supplementary captions data

**Data S1. Background of flocks.** The file provides information about the origin of the flocks, the number of chickens per pen, the breed and the date of hatch and arrival.

**Data S2. Details of loci with sequence variation in *C. gallinacea*.**

**Data S3. Comparison of translated CDSs.** Results of the local alignment approach in which all translated CDSs of *C. gallinacea* 08-1274/3 and *C. psittaci* NJ1, and vice versa were compared to each other to identify regions with less or no homology.

**Data S4. Unique CDSs.** List of CDS for which no homologue could be identified in *C. gallinacea* or *C. psittaci*. These data are graphically depicted in Fig 4B.

**Data S5. Results of analysis of predicted T3SS effectors.**

**Data S6. MLST and rMLST data.** In the file isolates used for rMLST and MLST including provenance and allelic profile data are listed with their allele numbers.
